# Importance of Endocytosis for the Biological Activity of Cedar Virus Fusion Protein

**DOI:** 10.3390/cells9092054

**Published:** 2020-09-08

**Authors:** Kerstin Fischer, Martin H. Groschup, Sandra Diederich

**Affiliations:** Institute of Novel and Emerging Diseases, Friedrich-Loeffler-Institut, Federal Research Institute for Animal Health, 17493 Greifswald—Insel Riems, Germany; Kerstin.Fischer@fli.de (K.F.); Martin.Groschup@fli.de (M.H.G.)

**Keywords:** cedar virus, henipavirus, fusion protein, endocytosis, biological activity

## Abstract

Endocytosis plays a particular role in the proteolytic activation of highly pathogenic henipaviruses Hendra (HeV) and Nipah virus (NiV) fusion (F) protein precursors. These proteins require endocytic uptake from the cell surface to be cleaved by cellular proteases within the endosomal compartment, followed by recycling to the plasma membrane for incorporation into budding virions or mediation of cell-cell fusion. This internalization largely depends on a tyrosine-based consensus motif for endocytosis present in the cytoplasmic tail of HeV and NiV F. Given the large number of tyrosine residues present in the F protein cytoplasmic domain of Cedar virus (CedV), a closely related but low pathogenic henipavirus, we aimed to investigate whether CedV F protein undergoes signal-mediated endocytosis from the cell surface controlled by tyrosine-based motifs present in its cytoplasmic tail and whether endocytosis is relevant for its biological activity. Therefore, tyrosine-based signals were mutated, and mutations were assessed for their effect on F cell surface expression, endocytosis, and biological activity. A membrane-proximal YXXΦ motif and a C-terminal di-tyrosine motif are of particular importance for cell surface expression and endocytosis rate. Furthermore, our data strongly indicate the pivotal role of endocytosis for the biological activity of the CedV F protein.

## 1. Introduction

Cedar virus (CedV) belongs to the *Henipavirus* genus within the *Paramyxoviridae* family and was first isolated from bat urine samples collected from an Australian *Pteropus* colony in 2012 [1]. Despite its genetic proximity to the highly pathogenic Hendra (HeV) and Nipah viruses (NiV), CedV has caused only asymptomatic infections in small animal models so far [1,2]. Therefore, research has focused on unraveling the molecular mechanisms leading to differences in the pathogenicity of these closely related viruses. One of the particularities of CedV is an impaired ability of the immunomodulatory phosphoprotein P to counteract the interferon response in cell culture [1,3]. Further differences are the receptor usage of the attachment proteins. The generally abundant expression of ephrins as cell entry receptors in numerous tissues and the high conservation among species results in a wide variety of susceptible hosts and a broad cell type tropism, which is fundamental to the zoonotic character and the pathogenesis of henipaviruses. While highly pathogenic HeV and NiV are known to utilize ephrin-B2, expressed i.e., in endothelial cells and lung tissue, and ephrin-B3, mainly found in the central nervous system, for cell entry [4,5,6,7], CedV is unable to use ephrin-B3 but rather binds to ephrin-B1, which is expressed in different tissues such as salivary glands, esophagus, and lung [8]. Therefore, recent studies have considered the distinct receptor usage of the CedV attachment protein to contribute to its reduced pathogenicity [8,9].

Besides receptor binding, the interaction of the attachment protein G with the viral fusion protein F is a prerequisite for virus entry into the host cell and virus spread. An indispensable step for the biological activity of fusion proteins and thus, viral infectivity, is the proteolytic cleavage of the precursor protein F_0_ into the two subunits F_1_ and F_2_ [10]. Interestingly, proteolytic activation of HeV and NiV F protein differs considerably from that of other paramyxoviruses in terms of subcellular localization and protease usage. After transport along the secretory pathway, newly synthesized HeV and NiV F protein precursors require endocytosis from the cell surface to encounter the activating host cell protease and then become biologically active. Cleavage within the endosomal compartment is then followed by recycling to the cell surface before the incorporation of mature fusogenic F_1_+F_2_ heterodimers into newly budding virions [11,12,13,14,15,16,17]. Overall, both viral envelope proteins are important determinants of pathogenicity that need to act in concert to promote virus-cell membrane fusion needed for virus entry as well as cell-cell fusion resulting in syncytia formation and thus, virus spread.

While trafficking through early and late endosomes prior to fusion with cellular membranes plays a critical role in virus entry of many viruses such as influenza virus [18,19], ebolaviruses [20,21], and flaviviruses [22,23], it is dispensable during NiV entry [16]. Moreover, other viruses and their glycoproteins hijack endosomal pathways in order to support their replication in infected cells [24,25]. The viral envelope glycoprotein of human immunodeficiency virus 1 (HIV-1) for instance undergoes endocytosis during the viral replication cycle, which is hypothesized to serve as a mechanism to evade the host immune response by reducing its cell surface expression (reviewed in [26,27]). In addition, trafficking of the HIV-1 envelope glycoprotein through the endocytic recycling compartment has been recently described as an essential step for incorporation into virus particles [28]. Interestingly, endocytosis of herpesvirus glycoproteins has been discussed to play a functional role in cell–cell fusion and in the production of infectious particles by delivering the glycoproteins to the intracellular site where virus assembly takes place [25,29,30]. Noteworthy, a recent report even suggests that endocytic trafficking of HeV F protein rather than its proteolytic cleavage is a crucial step for efficient HeV virus-like particle (VLP) assembly [31].

Apart from its importance for the viral replication cycle, endocytosis represents a key process for numerous cellular functions. Characterized by the internalization of the plasma membrane and extracellular molecules from the cell surface into internal membrane compartments, endocytosis is required for many biological events such as maintaining the plasma membrane composition or transporting selected cargo molecules from the cell surface to the interior [32]. Among the different types, clathrin-mediated endocytosis (CME) is the best characterized [33,34]. CME of a transmembrane protein is a highly coordinated process that primarily involves the interaction between the cytoplasmic domain of the protein and intracellular adaptor proteins (AP) that select transmembrane cargo into clathrin-coated pits via association to the clathrin lattice. These adaptor complexes recognize specific sorting motifs within the cytoplasmic tail of the cargo protein that are usually tyrosine- or leucine-based [35,36,37,38]. One typical sorting motif in cytoplasmic tails of transmembrane proteins known to interact with specific cytosolic adaptor complex proteins is the tyrosine-based YXXΦ motif, in which X can be any amino acid and Φ stands for an amino acid with a large hydrophobic residue. Numerous studies describe YXXΦ motifs to be recognized by AP2 that selects cargo from the plasma membrane on the one hand and facilitates binding to clathrin on the other hand culminating in CME of selected transmembrane cargo [35,36,37,38,39,40,41]. However, adaptor complex proteins are not only known to mediate endocytic uptake from the cell surface. They have also been reported to be involved in distinct transport pathways e.g., from early endosomes to recycling or late endosomes, as well as to lysosomes by adapting to specific motifs [38,42]. Clathrin-mediated endocytic uptake of HeV and NiV F proteins strongly depends on a characteristic YXXΦ motif located in the membrane-proximal part of the cytoplasmic tail as depicted in Table 1 [11,12,43]. Consequently, disruption of this motif led to measurable effects in the biological activity of the proteins [11,12] while another di-tyrosine motif in the C-terminal part of the tail had only negligible effects on NiV F endocytosis [11].

The cytoplasmic tail of CedV F protein displays several tyrosine residues that might act as functional endocytosis motifs. In addition to a C-terminal di-tyrosine motif similarly found in HeV and NiV F protein, a second di-tyrosine motif is present. Interestingly, two degenerate motifs of the YxxΦ are obvious: A YxxN motif that has been shown to be a functional endocytosis motif for the spike protein of the Porcine Epidemic Diarrhea Virus [44] and a YxxN motif that has been described to function as an endocytosis signal in the Measles virus hemagglutinin [45]. Thus, in this study, we aimed to investigate the impact of those tyrosine-based motifs on cell surface expression, endocytosis, and biological activity of the protein. Our results suggest a signal-mediated uptake of CedV F protein and confirm the functional importance of a membrane-proximal YXXΦ and a C-terminal di-tyrosine motif for the biological activity of CedV F protein.

## 2. Materials and Methods

### 2.1. Cell Lines, Transfection

Vero76, MDCK-2 and HeLa cells (Collection of Cell Lines in Veterinary Medicine, Friedrich-Loeffler-Institut, FLI; CCLV-RIE 0228, 1061 and 0082, respectively) were maintained in Dulbecco’s modified Eagle’s medium (DMEM) supplemented with fetal calf serum (10%; DMEM10) and incubated at 37 °C. Vero76 and MDCK-2 cells were reverse transfected by using the Lipofectamine 3000 reagent (Invitrogen, Schwerte, Germany) following the manufacturer’s protocol. HeLa cells were reverse transfected by using the METAFECTENE^®^ transfection reagent (Biontex, Munich, Germany) according to the manufacturer’s instructions.

### 2.2. Plasmids and Site-Directed Mutagenesis

The open reading frame (ORF) of CedV F and G (GenBank accession no. NC_025351.1) were synthesized by GeneArt (Thermo Fisher Scientific Inc., Regensburg, Germany) and subcloned into the pCAGGS expression vector. The ORF of the CedV F gene was codon-optimized for expression in human cells and HA-tagged at the C-terminus and will be designated as wild-type (wt) in the manuscript. Selected tyrosine residues within the cytoplasmic domain of CedV F protein were mutated to alanine by site-directed mutagenesis using the QuikChange Lightning Site-Directed Mutagenesis Kit (Agilent, Waldbronn, Germany) and primers designed according to manufacturer’s instructions.

### 2.3. Generation of Polyclonal Antibodies

To obtain a polyclonal antiserum raised against the CedV F protein, a rabbit was immunized subcutaneously three times at three-week intervals with CedV F protein. The antigen was produced in 293T cells as described earlier [46]. Briefly, plasmid DNA encoding for CedV F protein was used to transfect 293T cells. At 48 h post-transfection (p.t.), the supernatant was purified through a 20% sucrose cushion at 96,000× *g* for 2 h at 4 °C. After washing for 30 min at 155,000× *g*, pellets were resuspended in Tris-sodium chloride buffer. Immunization experiments were assessed and approved by the competent authority for animal welfare issues of the Federal State of Mecklenburg-Western Pomerania, Germany (license number: LALLF 7221.3-2-042/17).

### 2.4. Antibody Uptake Assay

MDCK-2 cells were reverse transfected with plasmids encoding for parental and mutant CedV F proteins (1 µg/well) in a 24 well plate as mentioned above. At 24 h p.t., confluent monolayers were washed and incubated without prior fixation with the polyclonal anti-CedV F rabbit serum (1:200 in 0.35% bovine serum albumin (BSA) in PBS^++^ (PBS with 0.5 mM MgCl, 1 mM CaCl_2_) (0.35% BSA/PBS^++^)). After incubation at 4 °C for one hour, cells were intensely washed and then either kept on ice or incubated with pre-warmed medium for 30 min at 37 °C to allow endocytic uptake. After fixing the cells with 4% paraformaldehyde (PFA), surface-bound primary antibodies were detected by an Alexa-Fluor (AF) 488-labeled secondary antibody (1:50 in 0.35% BSA/PBS^++^; LifeTechnologies, Darmstadt, Germany) for 90 min at 4 °C. After permeabilization with methanol-acetone (1:1) for 10 min, AF 568-labelled secondary antibodies (1:500 in 0.35% BSA/PBS^++^; LifeTechnologies, Darmstadt, Germany) were added to stain internalized primary antibodies. Images were acquired with an Eclipse Ti-S inverted microscope system and were processed with the ImageJ software version 1.45 s [47].

### 2.5. Metabolic Labeling

For pulse-chase analysis, MDCK-2 cells transiently expressing CedV F proteins were incubated at 24 h p.t. for 15 min with medium lacking cysteine and methionine, followed by incubation with medium containing [^35^S]cysteine and -methionine (Hartmann Analytic, Braunschweig, Germany) at a final concentration of 100 μCi/mL for 10 min (pulse). Then, labeling medium was replaced by a serum-free nonradioactive medium, and cells were incubated at 37 °C for 2 h (chase). Afterwards, cells were washed with PBS, followed by cell lysis in radioimmunoprecipitation assay (RIPA) buffer (1% Triton X-100, 1% sodium deoxycholate, 0.1% SDS, 0.15 M NaCl, 10 mM EDTA, 50 units/mL aprotinin, and 20 mM Tris-HCl, pH 7.5). Cell lysates were centrifuged for 45 min at 20,000× *g*, and CedV F proteins were immunoprecipitated using a polyclonal anti-HA antibody (H6908; 1:500; Sigma, Darmstadt, Germany). Protein A-Sepharose CL-4B (Sigma, Darmstadt, Germany) suspension was added for another 45 min followed by several washes of the immune complexes with RIPA buffer. After suspension in sample buffer, proteins were separated on a 12% polyacrylamide gel under reducing conditions. Dried gels were subjected to autoradiography and analyzed with a CR35 Dark Box Image analyzer (Duerr Medical, Bietigheim-Bissingen, Germany).

### 2.6. Colocalization Studies

For colocalization studies of CedV F and mutants with early endosomes, antibody uptake assays were performed as described above with modifications. Briefly, at 24 h p.t., CedV F-expressing HeLa cells were incubated with the polyclonal rabbit anti-CedV F serum (1:200) for 1 h at 4 °C. After washing, cells were shifted to 37 °C for 5 or 30 min to allow endocytosis to occur. Then, surface-bound primary antibodies were blocked with a horseradish peroxidase (HRP)-conjugated secondary antibody (1:50; LifeTechnologies, Darmstadt, Germany). After fixation with 2% PFA for 20 min and permeabilization with 0.2% Triton X-100 in PBS, a mouse anti-human early endosomal antigen 1 (EEA-1) antibody (1:50; BD Biosciences, Heidelberg, Germany) was added for 1 h at 4 °C for staining of early endosomes. Internalized primary rabbit antibodies were stained with AF 488-conjugated secondary antibodies (1:500; LifeTechnologies, Darmstadt, Germany). Primary mouse antibodies bound to EEA-1 were detected with AF 568-conjugated secondary antibodies (1:500; LifeTechnologies, Darmstadt, Germany). Cell nuclei were counterstained with 4′,6-Diamidin-2-phenylindol (DAPI). Representative images were recorded with a confocal laser scanning microscope (Leica SP5) and processed with the ImageJ software version 1.45 s [47].

### 2.7. Surface Biotinylation and Western Blot Analysis

MDCK-2 cells were transfected with plasmid DNA encoding either CedV F or mutant CedV F protein genes. At 24 h p.t., cells were washed and incubated twice with 2 mg/mL EZ-Link^®^ Sulfo-NHS-LC-Biotin (ThermoFisher Scientific, Waltham, MA, USA) for 20 min at 4 °C. Following cell lysis, F proteins were immunoprecipitated overnight using NeutrAvidin Agarose Resin (ThermoFisher Scientific, Waltham, MA, USA) according to the manufacturer’s protocol. Proteins were separated on a 10% SDS-gel under reducing or non-reducing conditions and then transferred onto nitrocellulose. Biotinylated CedV F proteins were detected by incubation with the HA-tag specific rabbit antibody H6908 (dilution 1:2,000 in PBS-Tween (0.05%)) followed by labeling with anti-rabbit HRP-conjugated secondary antibodies (1:5.000). Under non-reducing conditions, HRP-labeled Concanavalin A (ConA; Sigma-Aldrich, Darmstadt, Germany, 1:1,000 in PBS containing 0.05% (v/v) TWEEN 20, 1 mM CaCl_2_, 1 mM MnCl_2_, and 1 mM MgCl_2_ for 16 h at 20 °C) was used to stain NeutrAvidin-precipitated surface glycoproteins, which served as a loading control and as a reference to compare CedV F_0_ protein intensities. Proteins were visualized using a chemiluminescent substrate (Clarity Western ECL substrate, Bio-Rad, Feldkirchen, Deutschland) and the Bio-Rad Molecular Imager Chemi Doc^TM^ XRS+ in combination with the Image Lab software (Bio-Rad, Feldkirchen, Deutschland, Version 6.0.1).

### 2.8. Quantification of Endocytosis Rate by MESNA Reduction

MDCK-2 cells transiently expressing either CedV F or mutant F proteins were surface labeled with cleavable EZ-Link sulfo-NHS-SS-biotin (ThermoFisher Scientific, Waltham, MA, USA) at 4 °C. Then, wells were flooded with pre-warmed DMEM (37 °C) and shifted to 37 °C for either 5, 15, 30, or 90 min to allow endocytosis to occur. After rapid cooling to 4 °C, biotin still bound to the cell surface was cleaved by a membrane-impermeable reducing agent, 2-mercaptoethane-sulfonic acid sodium salt (MESNA; 50 mM in MESNA buffer (50 mM Tris, pH 8.5, 100 mM NaCl, 2.5 mM CaCl_2_)) a total of three times for 20 min. One sample was kept on ice and was neither incubated at 37 °C nor cleaved with MESNA and thus served as the surface biotinylation control to determine the total amount of biotinylated protein (control). After cell lysis in RIPA buffer, CedV F and CedV mutant F proteins were immunoprecipitated using the H6908 antibody as described above and separated by SDS-PAGE under non-reducing conditions. Transfer to nitrocellulose was followed by the detection of biotinylated proteins with HRP- labeled streptavidin and a chemiluminescent substrate. Protein bands were quantified using ImageLab software (version 6.0.1). Mean internalization rates per min were calculated as the ratio of band intensities from internalized, intracellular biotinylated protein after 5, 15, 30, and 90 min and total surface-labeled F protein (control, 50%) divided by 5, 15, 30, and 90 min, respectively.

### 2.9. Fusion Assay

A total of 4 × 10^5^ Vero76 cells or 3 × 10^5^ MDCK-2 cells were co-transfected with expression plasmids encoding for CedV G (0.5 µg/well) and either F or mutant F proteins (0.5 µg/well). At 48 h p.t., cells were fixed with ethanol and stained with 1:10-diluted Giemsa staining solution. Representative images (20× magnification) were recorded with an inverted microscope.

### 2.10. Luciferase Reporter Gene-Based Fusion Assay

Vero76 cells were reverse transfected with expression plasmids encoding for CedV G and either F or mutant F proteins as described above and seeded in 24-well plates. In addition, pCITE Renilla (200 ng/well), a vector containing the renilla luciferase gene under the control of the T7 promoter, was co-transfected. In parallel, Vero76 cells were reverse transfected with the pCAGGS T7 polymerase vector. At 27 h p.t., Vero76 cells expressing the T7 polymerase were washed twice with EDTA-containing PBS (5 mM), detached and added to the cells pre-transfected with pCAGGS CedV F and G and pCITE Renilla to allow fusion to proceed. After 3 h, cells were lysed using Lysis Buffer (pjk, GmbH, Kleinblittersdorf, Germany) and luciferase activity was measured by adding Renilla Glow substrate (pjk GmbH, Kleinblittersdorf, Germany) according to the manufacturer’s instructions. Samples were tested in duplicate in three independent experiments. Reporter activity detected upon co-transfection of the parental CedV F with CedV G protein was set to one, which served as a reference point for fusion activity. Reporter activities measured for the fusion of mutant CedV F proteins were used to calculate their fusion activity in relation to the parental protein. Background activity of the luciferase reporter was assessed with cells transfected with pCAGGS CedV G and pCITE Renilla only overlaid with T7 polymerase expressing cells.

## 3. Results

**Figure 1:**
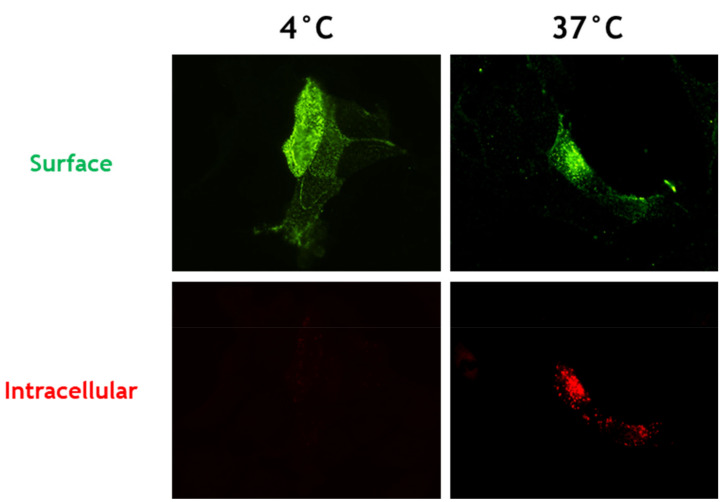
Endocytosis of CedV F protein in MDCK-2 cells. At 24 h p.t., CedV F-expressing MDCK-2 cells were incubated with a CedV F-specific rabbit antiserum to label surface-expressed F proteins. Then, the cells were shifted to 37 °C for 30 min to allow endocytosis to occur. AlexaFluor (AF) 488-conjugated secondary antibodies visualized surface-bound primary antibodies. After fixation and permeabilization, AF 568-conjugated secondary antibodies were used to stain internalized primary antibody-CedV F protein complexes. Magnification, ×60. n = 3.

Endocytosis of HeV and NiV F protein precursors represents a critical step to gain biological activity, and thus, viral infectivity. In order to investigate whether CedV F protein similarly undergoes endocytosis from the cell surface, we performed a qualitative antibody uptake assay as described previously [11]. Therefore, MDCK-2 cells were transfected with plasmid DNA encoding for the CedV F protein. At 24 h p.t. without prior fixation, cell-surface expressed CedV F protein was labeled with CedV F protein-specific antibodies. Next, cells were either kept on ice or shifted to 37 °C for 30 min to allow endocytosis to occur. Surface-bound antibodies were then detected with an AF 488-conjugated secondary antibody that was added in excess to saturate surface-bound primary antibodies. Subsequent permeabilization allowed the staining of intracellular F protein-antibody complexes with an AF 568-conjugated antibody. After incubation at 37 °C, cells expressing the CedV F protein showed both green surface staining and multiple red fluorescent intracellular vesicles indicating endocytosis of the labeled CedV F protein (Figure 1). In contrast, we observed only green fluorescent signals of F proteins in cells that were kept on ice suffering a temperature-related inhibition of endocytosis.

**Figure 2:**
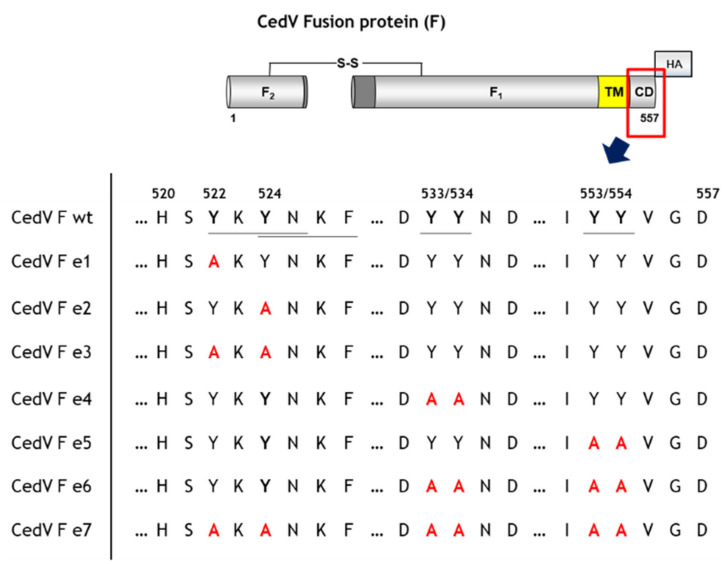
Schematic overview of the cytoplasmic tail mutants of CedV F protein generated in this study. Tyrosine-based putative endocytosis motifs are underlined. F_1_: CedV F protein subunit F_1_; F_2_: CedV F protein subunit F_2_; TM: transmembrane domain; CD: cytoplasmic domain; HA: HA-tag at the C-terminus of the F_1_ subunit; S-S: di-sulfide bond. wt: wild-type.

To evaluate the functional importance of the tyrosine-based motifs within the CedV F protein cytoplasmic tail for endocytosis, we generated seven F protein mutants by tyrosine to alanine substitution (Figure 2). We constructed mutants encoding either single (e1–e5) or multiple (e3, e6) mutations as well as one mutant devoid of all putative endocytosis signals (e7) to investigate potentially additive effects of these mutated motifs (Figure 2). Apart from a classical YXXΦ motif in the membrane-proximal region (Y_524_XXF, Figure 2), we chose to also mutate the tyrosine residue at amino acid position 522 (Y_522_XXN, Figure 2) since a similar motif present in the cytoplasmic domain of Measles virus hemagglutinin has been described earlier to affect basolateral sorting and internalization of the protein [45].

**Figure 3:**
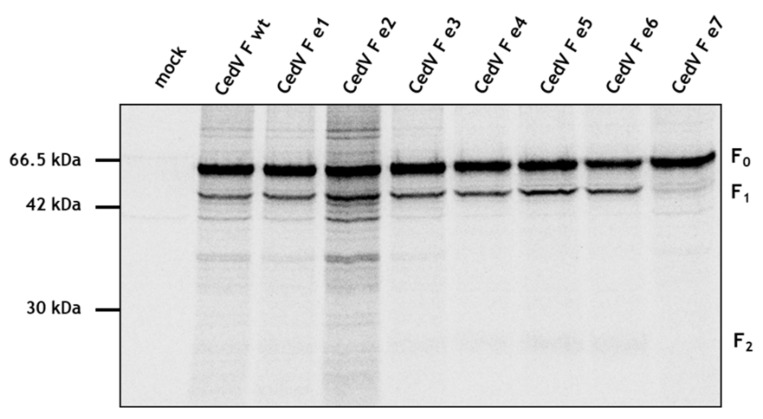
Effect of cytoplasmic tail mutations on CedV F protein expression and cleavage. At 24 h p.t., MDCK-2 cells expressing different CedV F proteins were metabolically labeled for 15 min (pulse) and then incubated for 2 h in serum-free nonradioactive medium (chase). After immunoprecipitation of F proteins from cell lysates and separation on a 12% SDS-gel under reducing conditions, samples were analyzed by autoradiography. n = 2; wt: wild-type.

To assess whether these mutations have an impact on F protein synthesis, we compared expression efficiencies using a pulse-chase analysis. At 24 h p.t., MDCK-2 cells were labeled metabolically with [35] cysteine and -methionine for 15 min. After pulse-labeling, cells were incubated for 2 h followed by immunoprecipitation and SDS-PAGE under reducing conditions. We observed similar amounts of expression for parental and mutant F proteins within 2 h demonstrating that mutations within the cytoplasmic tail do not impair protein synthesis (Figure 3). However, although mutant e7 was expressed at similar levels, proteolytic processing was much less efficient. We generated additional F protein constructs combining selected mutations of particular motifs (e8 = e3 + e4; e9 = e3 + e5). Since these mutants displayed no differences in proteolytic processing to F wt (Supplementary Figure S1), they were not included in subsequent analyses. Together, these findings suggest that proteolytic activation of the CedV F protein is markedly impaired upon the simultaneous disruption of all tyrosine-based motifs.

**Figure 4:**
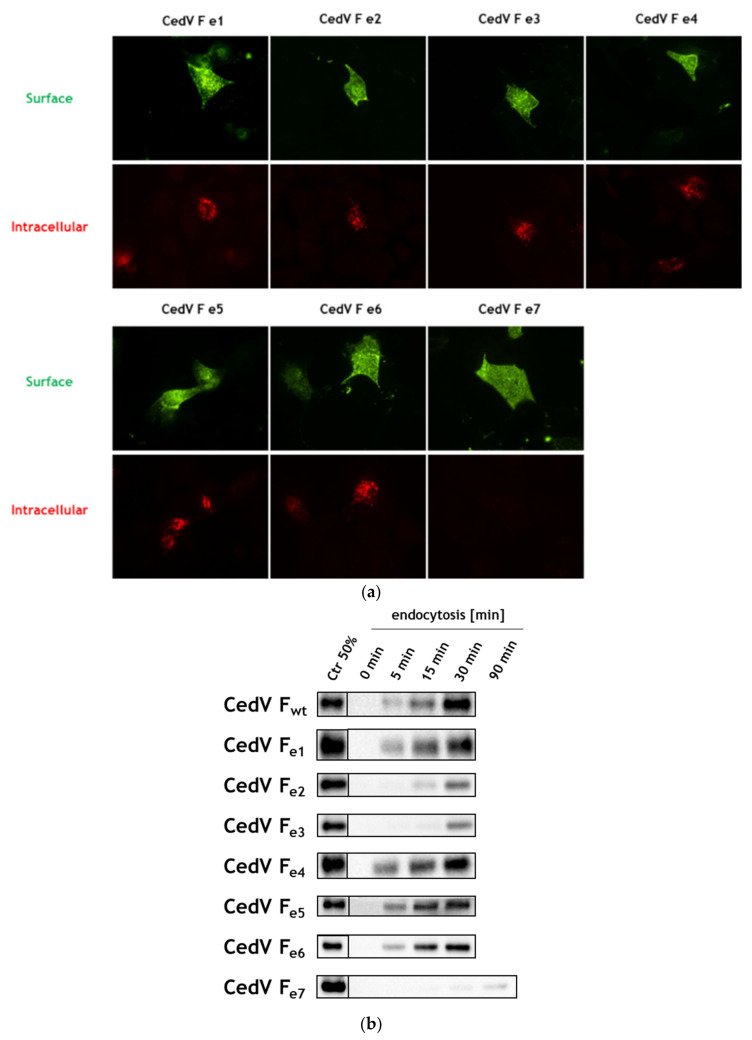

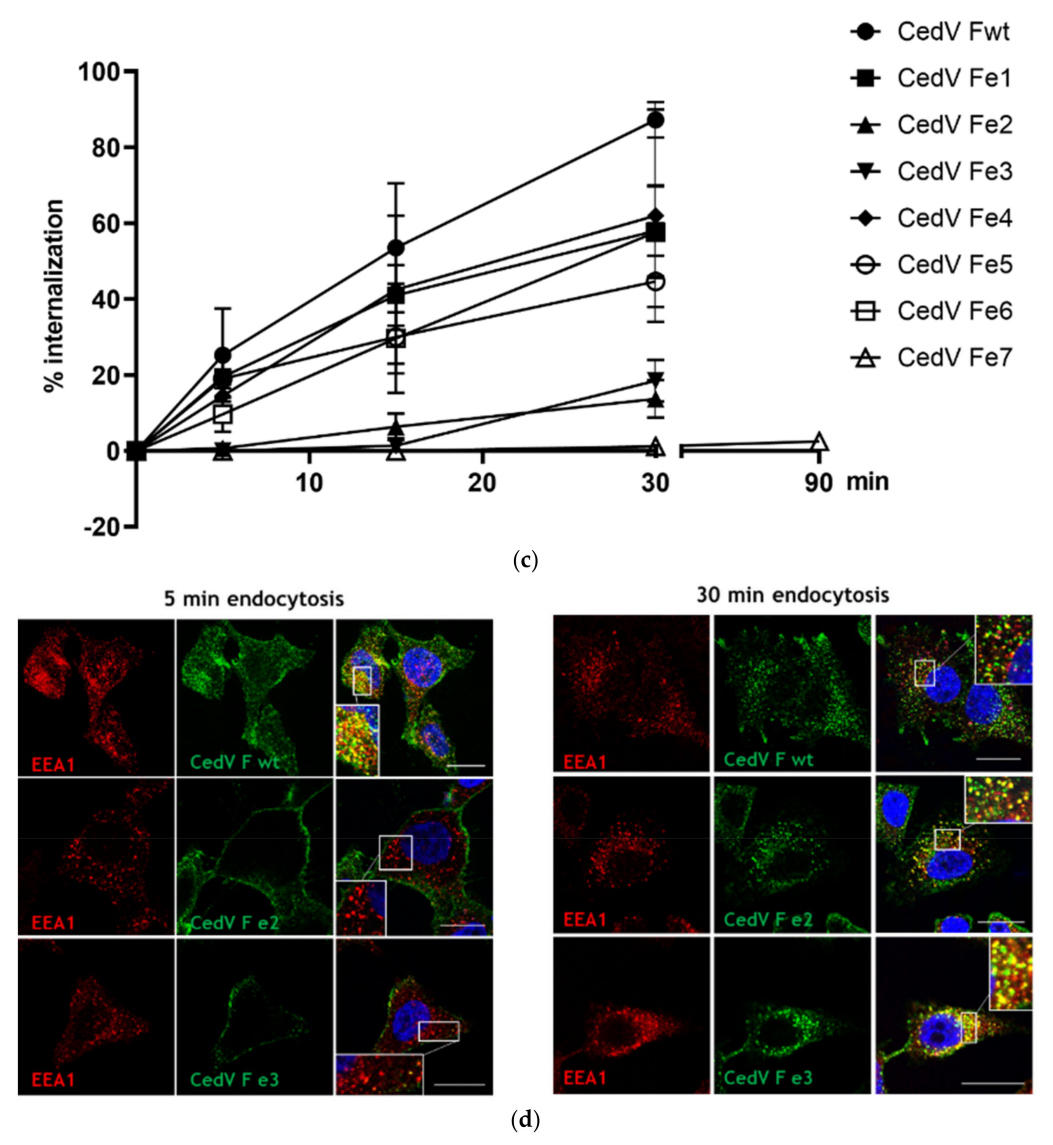
Endocytosis of CedV F proteins in MDCK-2 cells. (**a**) Antibody uptake assay of mutant CedV F proteins. MDCK-2 cells were transfected with plasmids encoding the indicated CedV F protein mutants. At 24 h p.t., a CedV F-specific rabbit antiserum was used to label surface-expressed F proteins at 4 °C. Then, the cells were shifted to 37 °C for 30 min to allow endocytosis to occur. AF 488-conjugated secondary antibodies visualized surface-bound primary antibodies. After fixation and permeabilization, AF 568-conjugated secondary antibodies were used to stain internalized primary antibody-CedV F protein complexes. Magnification, ×60. n = 2. (**b**) At 24 h p.t., CedV F-expressing cells were surface-labeled with cleavable NHS-SS biotin at 4 °C followed by a shift to 37 °C for the indicated times allowing endocytosis to occur. Residual biotin was then cleaved from the cell surface using MESNA. To quantify the amount of surface-biotinylated proteins that underwent endocytosis in a certain time, samples were compared to the total amount of surface-biotinylated control cells (Ctr) that were neither incubated at 37 °C nor treated with MESNA. Following cell lysis, F proteins were immunoprecipitated and samples were separated under non-reducing conditions. After transfer to nitrocellulose, biotinylated proteins were detected with peroxidase-conjugated streptavidin and chemiluminescence. The control lane represents 50% of the total amount of biotinylated F proteins (Ctr 50%). One representative blot is shown for each CedV F protein variant. (n = 3; n = 2 for mutants e1, e3, and e4). wt: wild-type (**c**) CedV F protein internalization in percentage (%) per minute. To quantify the internalization rate, the percentages of internalized F protein amounts measured in the experiment shown in Figure 4b are displayed as a function of the time of incubation at 37 °C. Error bars represent the standard error of the mean. (**d**) Intracellular localization of wt and mutant CedV F proteins in MDCK-2 cells after 5 and 30 min at 37 °C. An antibody uptake assay of CedV F proteins was performed as described above with slight modifications. After the endocytosis step, surface-bound primary antibodies were blocked using a peroxidase-labeled secondary antibody while internalized primary antibodies were detected with AF 488-conjugated rabbit-specific secondary antibodies after fixation and permeabilization. Likewise, early endosomes were visualized with a primary antibody against the early endosomal antigen-1 (EEA1) and a mouse-specific AF568-conjugated secondary antibody. Scale bars indicate 20 µm. Representative images from two independent experiments are displayed (n = 2). Inserts show magnifications of indicated areas. Magnification, ×63.

HeV and NiV F proteins are known to be cleaved within the endosomal compartment. In analogy, blocking clathrin-mediated endocytosis by addition of chlorpromazine also inhibited proteolytic processing of CedV F protein (see Supplementary Figure S2) demonstrating that endocytosis is a prerequisite for CedV F cleavage. Thus, inefficient cleavage of CedV mutant e7 might result from an inability to undergo endocytosis. In order to study the effects of single and multiple cytoplasmic tail mutations on CedV F protein endocytosis, we performed another antibody uptake experiment as described above using all generated mutants. After a period of 30 min at 37 °C, we observed intracellular red vesicles for all mutants except mutant e7 with all tyrosine-based motifs disrupted (Figure 4a). Disruption of a single motif such as YXXN (mutant e1) or YXXF (mutant e2), the di-tyrosine motifs (mutants e4, e5) as well as the combination of several mutated tyrosine residues (mutants e3, e6) displayed no difference in comparison to the fluorescent signals of the parental CedV F protein (Figure 1). The qualitative finding of red fluorescent vesicles indicates the internalization of mutants e1 to e6 from the cell surface while endocytosis of mutant e7 was strongly impaired after 30 min of endocytosis.

To further understand the effects of single and multiple mutations on endocytosis, we next aimed to quantify the internalization of the different CedV F proteins and compare their endocytosis rate with the wt CedV F protein in a semi-quantitative biotin internalization assay. Moreover, this assay allowed us to demonstrate that the internalization of F proteins is not induced by antibody cross-linking. Briefly, we performed a surface biotinylation assay using a cleavable NHS-SS biotin derivative. Using the membrane-impermeable reducing agent MESNA, residual biotin that was not internalized from the cell surface after incubation at 37 °C for different periods can be cleaved. Cell lysis, immunoprecipitation of F proteins, and Western blot using Streptavidin-HRP were performed with subsequent detection and quantification of intracellular biotinylated F proteins. The amount of internalized protein was quantified by comparison to F-expressing cells that were neither incubated at 37 °C nor treated with MESNA, therefore, representing the total amount of biotinylated F proteins (Ctr lane, Figure 4b). Since internalization rates did not appear to be linear over time, endocytosis of biotinylated proteins is displayed additionally as a function of the time in Figure 4c. More than 80% of the parental CedV F protein was internalized after 30 min (Figure 4b,c). Similarly, mutant e1 carrying a substitution of a single tyrosine residue at aa position 522 as well as the di-tyrosine motif mutants e4, e5, and e6 displayed only a marginal decrease in internalization compared to the parental F protein. Interestingly, mutation of the YXXΦ motif (mutant e2 and e3) clearly decreased internalization with weak signals detectable after a period of 15 to 30 min at 37 °C (Figure 4b). However, the strongest effects were observed for mutant e7. Here, biotinylated mutant e7 was only detected after 90 min of incubation at 37 °C (Figure 4b) leading to a drastically reduced internalization rate over time in contrast to the parental F protein and the other mutants (Figure 4c). An antibody uptake assay of CedV F proteins including a co-staining of endosomal marker protein early endosomal antigen-1 (EEA1) in HeLa cells confirmed the delayed uptake of mutants e2 and e3. After 5 min at 37 °C, we observed no internalization of CedV F mutants e2 and e3 and thus, no co-localization with EEA1 (Figure 4d). However, both mutants co-localized with EEA1 after 30 min. In contrast, the parental F protein showed intracellular staining and co-localization with EEA1 as early as 5 min after the shift to 37 °C (Figure 4d).

**Figure 5:**
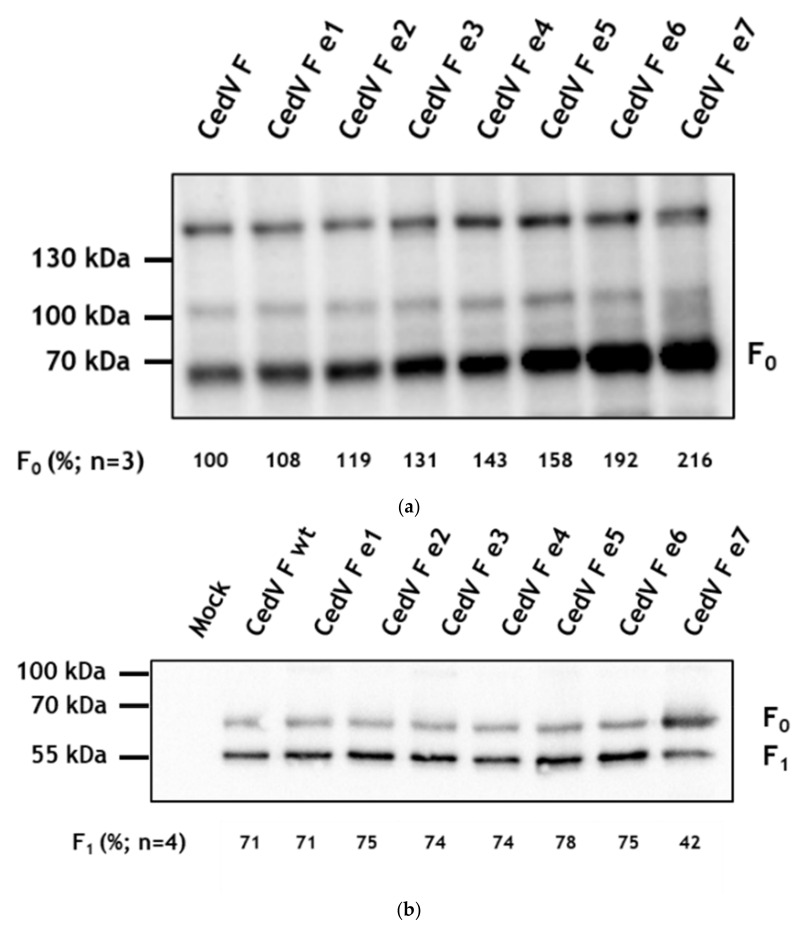
Cell surface expression of CedV F proteins. At 24 h p.t., MDCK-2 cells expressing F proteins were surface-labeled with biotin on ice. After cell lysis, biotinylated proteins were immunoprecipitated using NeutrAvidin beads and subjected to SDS-PAGE under non-reducing (**a**) and reducing (**b**) conditions. Precipitated F proteins were visualized using an antibody against the HA-tag (H6908), HRP-labeled secondary antibodies, and chemiluminescence. In (**a**), ConA staining is used as a loading control. Representative blots are shown from (**a**) three or (**b**) four independent experiments. The amount of F_0_ and F_1_ protein (in (**a**) % of CedV F_0_ protein with the parental F protein set to 100% or (**b**) % of F_1_ protein) is calculated as the mean of three or four independent experiments, respectively. wt: wild-type.

Since differences in the internalization rate might directly affect cell surface expression, we next performed a surface biotinylation assay of F-expressing MDCK-2 cells followed by immunoprecipitation of biotinylated proteins and detection of the HA-tagged proteins in Western blot. In accordance with the antibody uptake assay and the MESNA reduction, we observed that all F proteins reached the cell surface (Figure 5). However, comparing the cell surface expression of all F proteins under non-reducing conditions, the amount of F_0_ differed distinctly between the parental CedV F protein and some mutant F proteins (Figure 5a). In all biotinylation assays performed mutant e1 displayed a cell surface expression similar to the parental CedV F protein, while surface expression of mutants e2–e5 was slightly increased. In contrast, the average level of cell surface expression of mutants, e6 and e7 was substantially higher. For mutant e7, we detected up to 2.5-fold more F_0_ on the cell surface than for the parental F protein. Under reducing conditions (Figure 5b), both the F_0_ precursor and the subunit F_1_ were detected for all F proteins analyzed, indicating that all of them were proteolytically cleaved despite mutations in their cytoplasmic tails. For the parental F protein as well as for mutant e1–e6, quantification of band intensities revealed that more cleavage product F_1_ than inactive precursor F_0_ is found at the cell surface. However, mutant e7 rather displayed a reversed cleavage ratio, with less F_1_ present on the cell surface indicating a reduced amount of cleaved F protein. Although internalization of mutants e2 and e3 was shown to be clearly delayed in the biotin internalization assay, proteolytic activation and surface expression of these mutants still seemed to reach levels comparable to the parental F protein after 24 h p.t.

**Figure 6:**
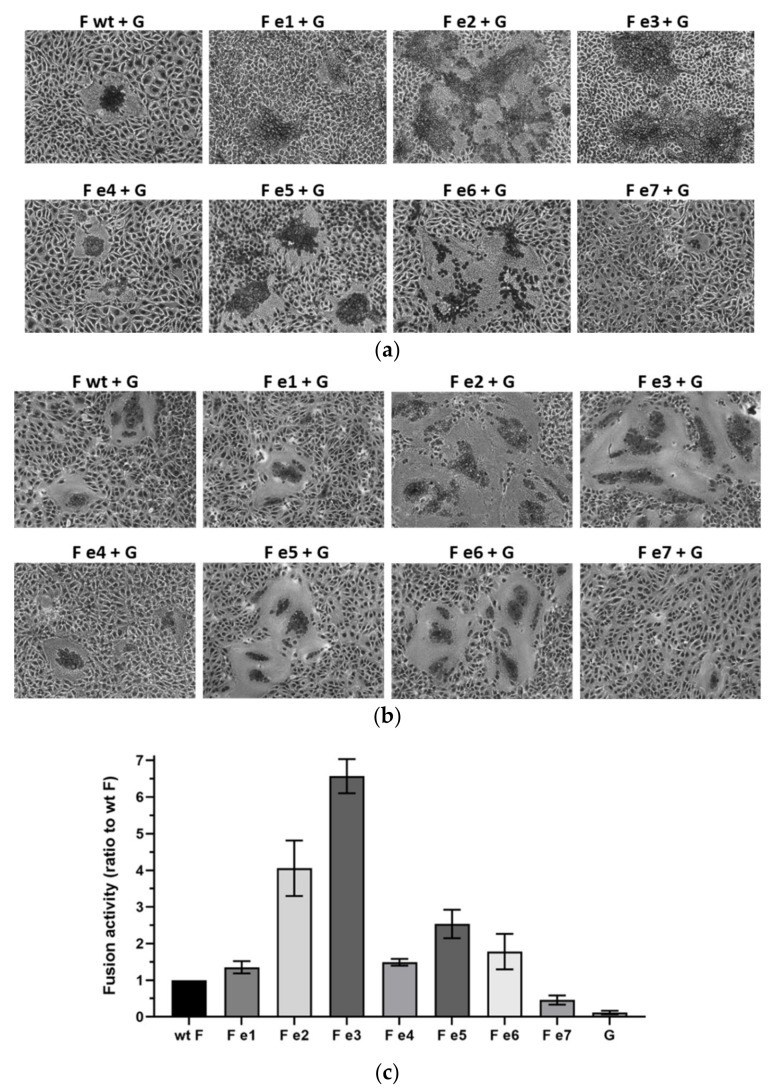
Effect of cytoplasmic tail mutations on CedV glycoprotein-mediated fusion activity. Syncytium formation in (**a**) MDCK-2 and (**b**) Vero76 cells co-expressing CedV F and G proteins were visualized by Giemsa staining at 48 h p.t. Magnification, ×20. n = 3; wt: wild-type (**c**) Quantitative reporter gene assay. Vero76 cells were co-transfected with plasmids encoding for the CedV glycoproteins F and G as well as a plasmid containing the luciferase gene under the control of a T7 promoter (pCITE Renilla). At 24 h p.t., Vero76 cells expressing the T7 polymerase were layered on the glycoprotein-expressing cells and incubated for 3 h at 37 °C. Then, cells were lysed, and luciferase activity measured using a luminometer. Samples were tested in duplicates in three independent experiments. Reporter activity measured for the parental CedV F protein (wt F) co-transfected with CedV G protein was set to 1 serving as a reference point for fusion activity. Bars represent the fusion activities of the different (mutant) CedV F proteins in relation to the fusion activity of the parental protein and include the standard error of the mean (SEM). Background activity of the luciferase reporter was assessed with cells transfected with pCAGGS CedV G and pCITE Renilla only, layered with T7 polymerase expressing cells. n = 3.

Finally, to assess whether any of the observed effects influence the biological activity of the F proteins, syncytium formation was analyzed in a fusion assay. At 48 h p.t., co-expression of the parental CedV F and CedV G proteins resulted in the formation of multinucleated syncytia in MDCK2 cells (Figure 6a) and Vero76 cells (Figure 6b). Mutant CedV F e1 and e4 induced syncytium formation comparable to the parental F protein. Surprisingly, despite their reduced endocytosis rate but parental F-like cell surface expression, co-expression of mutant e2 or e3 with CedV G led to a hyperfusogenic phenotype forming syncytia that were markedly increased in number and size (Figure 6a,b). Interestingly, fusogenicity of mutant e5 and e6 displaying an increased surface expression compared to the parental F protein was slightly enhanced while fusion activity of endocytosis-deficient mutant e7 was strongly impaired in both cell lines tested (Figure 6a,b). In addition, we quantified these differences using a luciferase reporter gene-based fusion assay in Vero76 cells after 24 h p.t. The hyperfusogenic phenotype of mutant e2 and e3 displayed a 4- and 6.5-fold increase in measurable luciferase reporter activity compared to the parental F protein (Figure 6c). Co-expression of CedV G protein with mutant e5 still resulted in a 2.5-fold increase in reporter activity in Vero76 cells (Figure 6c). A marked decrease in reporter activity was confirmed for mutant e7. Since fusion activity is sensitive to the cell surface expression of both glycoproteins and to exclude that observed effects in fusogenicity were due to an altered cell-surface expression of CedV G protein, we performed a surface biotinylation assay in MDCK-2 cells co-transfected with CedV G and wt or mutant CedV F proteins. As depicted in Supplementary Figure S3, both F and G can be detected at the cell surface. Irrespective of the (mutant) F protein combination, the G expression at the cell surface seems to be similar suggesting that observed effects in fusion activity are not related to differences in CedV G cell surface expression. Taken together, these findings demonstrate that a membrane-proximal YXXΦ, as well as a C-terminal di-tyrosine motif in the CedV F protein cytoplasmic tail, are of functional relevance for endocytosis and biological activity of the protein.

## 4. Discussion

Endocytic uptake from the cell surface plays a particular role in the replication cycle of highly pathogenic HeV and NiV [11,12,13]. Previous work has shown the importance of endocytosis for the maturation of F proteins to gain biological activity and thus, viral infectivity [11,12,13,16]. Given the fact that endocytic uptake of HeV and NiV F proteins depends on tyrosine-based motifs present in their cytoplasmic tail, the comparably high number of tyrosine residues found in the CedV F protein was of interest for this study. We first aimed to investigate whether these residues indeed mediate endocytosis of the protein. Second, we analyzed whether endocytosis is of functional relevance to the CedV F protein maturation. Taken together, our data clearly indicate that endocytic uptake of CedV F protein is signal-mediated and important for the biological activity of the protein. Furthermore, among the putative endocytosis signals, we identified a YXXΦ motif and a C-terminal di-tyrosine motif to have the strongest effects on endocytosis, cell surface expression, and fusion activity of CedV F protein.

The YXXΦ motif known to facilitate clathrin-mediated endocytosis of many transmembrane proteins is present in the membrane-proximal region of the cytoplasmic tail of both high and low pathogenic henipavirus F proteins. Mutation of the YXXΦ motif in the F protein of low pathogenic CedV resulted in a strongly decreased internalization rate for mutant e2 and e3, suggesting endocytic uptake of CedV F protein to be signal-mediated and largely dependent on this particular motif and most likely on Y524. For highly pathogenic HeV and NiV, several studies have similarly shown that an intact YXXΦ motif greatly contributes to the internalization of F proteins with a marked reduction in endocytosis rate upon motif disruption [11,12,13,43]. However, while the delayed endocytic uptake of NiV F protein led to a reduced fusogenicity of the mutant, the respective mutation in the HeV F protein rather resulted in an enhanced cell-to-cell fusion upon interaction with the viral attachment protein G [11,12]. Due to strong reduction but not the absence of endocytosis, the authors suggested an accumulation of recycled, fusogenic HeV F protein at the cell surface over time to account for the observed effects. For the respective CedV F mutants, we similarly found a clear delay in internalization (Figure 4b), but only moderate differences in total surface expression compared to the parental CedV F protein (Figure 5). However, we did indeed measure a strong 4- to 6.5-fold increase in the fusion activity of these two mutants in contrast to the parental CedV F protein, which cannot exclusively be explained by the only slightly increased cell surface expression. Although triggering of fusion is usually thought to primarily involve the ectodomain of the fusion protein, hyper- and hypofusogenicity were also observed in NiV F cytoplasmic tail mutants due to specific mutations in a membrane-proximal polybasic KKR motif [48]. The authors explained their findings by a mutation-dependent inside-out signaling mechanism resulting in conformational changes in the NiV F ectodomain followed by measurable effects on the fusion activity of the protein [48]. Overall, such conformational changes of the F-ectodomain due to mutations in the cytoplasmic tail can also affect the avidity of F and G interaction and thus, the coordinated processes required for cell–cell fusion [48,49,50]. Further, it is believed that mechanisms resulting in virus entry (viral—cellular membrane fusion) and cell–cell-fusion (fusion of neighboring cell membranes) are closely related [51]. Thus, cell–cell fusion levels often correlate to viral entry levels as shown for several Hendra and Nipah glycoprotein mutants [48,52,53,54]. In contrast, some hyperfusogenic G mutants with a modified O-glycosylation were described to display reduced entry levels but similar cell–cell fusion levels [55]. Also, a headless NiV G mutant readily triggered cell–cell fusion but pseudotyped NiV virions did not enter cells [56]. Hence, the underlying mechanism for the observed hyperfusogenicity of the CedV F mutants e2 and e3 and here the potential role of Y524 will have to be addressed in future studies.

Apart from a functional YXXΦ motif, the cytoplasmic tail of CedV F protein contains two additional di-tyrosine based motifs. Mutation of the di-tyrosine motif at amino acid position Y533/534A (mutant e4) showed no effect in any of the assays performed. However, mutation of the C-terminal di-tyrosine motif (Y553/554A; mutant e5 and e6) led to a detectable increase of cell surface expression with slightly enhanced fusion activity. On the one hand, this phenotype could result from a marginally reduced internalization rate. Alternatively, this di-tyrosine motif may affect the dynamics of endosomal trafficking and recycling of CedV F proteins, which could subsequently alter the availability of fusion-active F protein on the cell surface, thus having an impact on fusion activity. In a previous study, the lack of the di-tyrosine motif has been considered to delay the recycling of fusogenic NiV F proteins to the cell surface [43]. A tail-truncated NiV F variant lacking the C-terminal di-tyrosine motif was markedly downregulated in constitutive surface expression while F protein endocytosis and endosomal cleavage remained unaffected. Consequently, the intact di-tyrosine motif was hypothesized to act as a potential cytoplasmic recycling motif affecting intracellular trafficking [43]. Interestingly, both the YXXΦ and the C-terminal di-tyrosine motif have been described to be of importance for NiV F protein trafficking and sorting in polarized microvascular endothelial cells [57], in polarized epithelial cells [58], and in polarized neuronal cells [59].

The presence of intact tyrosine-based endocytosis and/or sorting motif in the cytoplasmic tail of transmembrane proteins has been described to be important for the replication cycle, infectivity, and virulence of many viruses such as herpes-, corona- and retroviruses [24,25,29,30,44,60,61]. Apart from signal-mediated intracellular trafficking of viral proteins to the sites of viral assembly, these motifs were shown to affect cell surface expression of different viral transmembrane proteins [24,25,29,30,44,60,61]. For instance, mutating the endocytosis signal of the envelope protein of simian immunodeficiency virus led to more efficient incorporation of envelope proteins into budding virions with enhanced infectivity due to increased levels of envelope protein expressed on the cell surface [61]. In the case of the CedV F protein, mutation of the di-tyrosine motif had stronger effects on the cell surface expression than mutation of the classical YXXΦ motif. However, at this point, it is not clear whether the di-tyrosine mutants will be incorporated more efficiently into virions due to an increase in cell surface expression. Mutation of the di-tyrosine motif in the NiV F cytoplasmic tail almost completely abrogated NiV VLP budding despite the wt-like cell surface expression of the protein [62]. Additionally, the presence of an intact and functional YXXΦ endocytosis motif in the cytoplasmic tail of NiV and HeV F protein has been considered to play a critical role in endocytic trafficking and recycling, and thus, in efficient viral assembly and particle release [31,62]. Similar findings have been reported for the envelope protein of HIV-1 where recent evidence suggests that trafficking through the recycling endosome is required for efficient incorporation of viral envelope proteins into virus particles [28]. Considering the lack of an intact YXXΦ motif, the reduced endocytosis rate, and the level of cell surface expression similar to the parental F protein, it will be interesting to see whether assembly and budding of mutant CedV F e2 and e3 is impaired or rather enhanced.

The most significant effects on cell surface expression, internalization rate, and fusion activity were noted for CedV mutant e7, in which all putative tyrosine-based endocytosis motifs were mutated. Importantly, the combination of all motif mutations led to an almost abrogated fusion activity of the protein with the strongest delay in internalization, which is in accordance with previous findings for a NiV F protein mutant disrupted of all tyrosine-based motifs [11]. Though CedV F mutant e7 displayed the highest cell surface expression, the combination of all mutations led to a rather reversed cleavage ratio in contrast to the other mutants. These findings point towards a reduced proteolytic F activation, which might result from the marked decrease in the endocytosis rate. Considering what is known for the F proteins of highly pathogenic HeV and NiV that are cleaved within the endosomal compartment [12,13,14,15,17], it seems likely that the proteolytic activation of CedV F protein is quite similar in terms of subcellular localization. However, future studies are needed to unravel shared commonalities and potential differences in this process.

## 5. Conclusions

In conclusion, our data indicate that CedV F protein is indeed internalized from the cell surface as described for HeV and NiV F proteins. Furthermore, endocytic uptake of CedV F protein is signal-mediated and represents a key step in order to gain biological activity. Future studies should investigate proteolytic activation in more detail in order to find further commonalities and/or differences in subcellular localization and protease usage between high and low pathogenic henipaviruses. Further, tyrosine-based motifs should be analyzed for their effects on sorting and endocytic trafficking of the CedV F protein including their ability to influence virus particle assembly and their potential effect on virus-cell fusion/infectivity.

## Figures and Tables

**Table 1:** Boldface and underlined letters highlight (potential) endocytosis signals. Numbers above the sequence indicate the amino acid position. Cytoplasmic tails for NiV and HeV F proteins range from amino acid positions 519 to 546, cytoplasmic tail for CedV F is predicted for amino acid positions 517 to 557. Basic aa’s that have been shown to be of importance for fusion protein functionality are highlighted in orange.

	519	546
**NiV F**	…EKKRNT**YS****R****L**EDRRVRPTSSGDL**YY**IGT	
**HeV F**	…EKKRGN**YS****R****L**DDRQVRPVSNGDL**YY**IGT	
	**517**	**557**
**CedV F**	…KSKHS****Y****K****YN****K****F****IDDPD****YY****NDYKRERINGKASKSNNI****YY****VGD	

## Supplementary Materials

The following are available online at https://www.mdpi.com/2073-4409/9/9/2054/s1, Figure S1: Proteolytic processing of CedV F wt and mutants e8 (e3 + e4) and e9 (e3 + e5), Figure S2: Proteolytic processing of CedV F protein is inhibited by chlorpromazine, Figure S3: Co-expression of CedV F wt, mutants and CedV G protein on the cell surface.
