# Supplementary material for: Importance of Endocytosis for the Biological Activity of Cedar Virus Fusion Protein
Source: Cells. 2020 Sep 8;9(9):2054. doi: 10.3390/cells9092054 (PMC7565975; doi:10.3390/cells9092054)
Supplement: Supplementary file 1 [file cells-09-02054-s001.pdf]

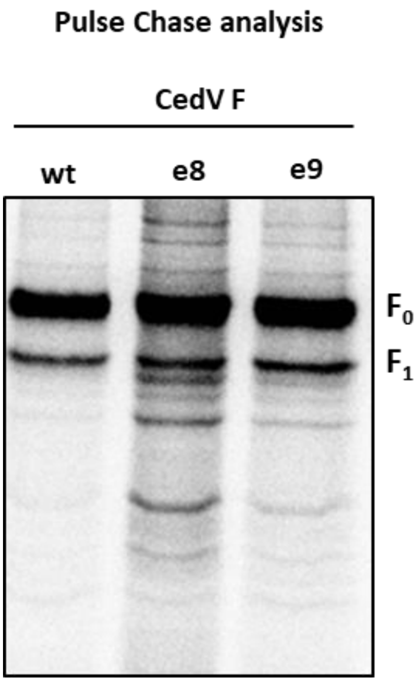

Figure S1: Proteolytic processing of CedV F wt and mutants e8 (e3 + e4) and e9 (e3 + e5). At 24 h p.t., MDCK-2 cells expressing different CedV F proteins were metabolically labeled for 15 min (pulse) and then incubated for 2 h in serum-free nonradioactive medium (chase). After immunoprecipitation of F proteins from cell lysates and separation on a 12% SDS-gel under reducing conditions, samples were analyzed by autoradiography; n = 2; wt: wild-type

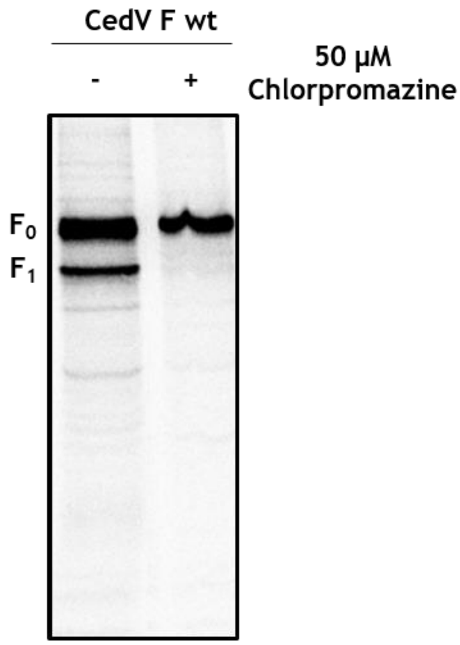

Figure S2: Proteolytic processing of CedV F protein is inhibited by chlorpromazine. At 24 h p.t., MDCK-2 cells expressing CedV F protein were metabolically labeled for 15 min (pulse) and then incubated for 3 h in serum-free nonradioactive medium in the absence (-) or presence (+) of 50 µM chlorpromazine (chase). After immunoprecipitation of F proteins from cell lysates and separation on a 12% SDS-gel under reducing conditions, samples were analyzed by autoradiography; wt: wild-type

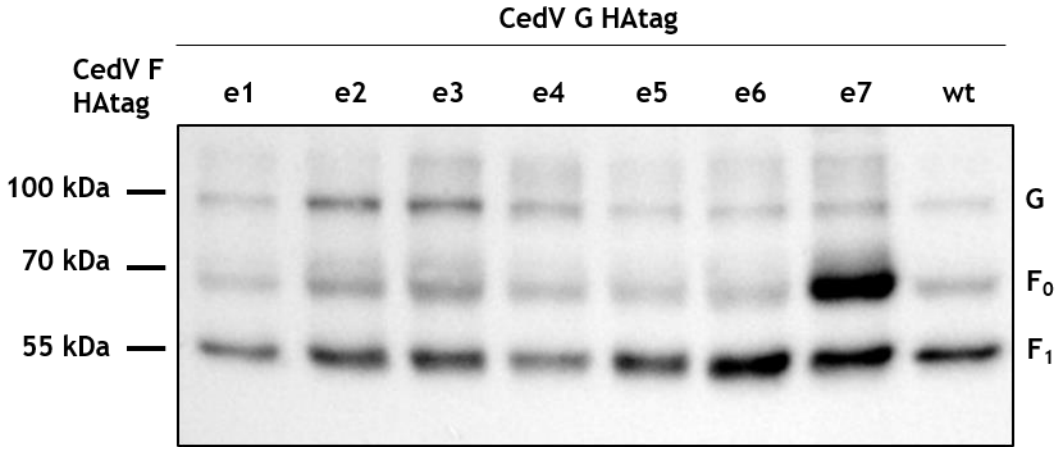

Figure S3: Co-expression of CedV F wt, mutants and CedV G protein on the cell surface. At 24 h p.t., MDCK-2 cells co-expressing different CedV F proteins and CedV G protein were surface-labeled with biotin on ice. After cell lysis, biotinylated proteins were immunoprecipitated using NeutrAvidin beads and subjected to SDS-PAGE under reducing conditions. Precipitated F proteins were visualized using an antibody against the HA-tag (H6908), HRP-labeled secondary antibodies and chemiluminescence. n = 2; wt: wild-type.
